# Relationship between Fusarium Head Blight, Kernel Damage, Concentration of *Fusarium* Biomass, and *Fusarium* Toxins in Grain of Winter Wheat Inoculated with *Fusarium culmorum*

**DOI:** 10.3390/toxins11010002

**Published:** 2018-12-21

**Authors:** Tomasz Góral, Halina Wiśniewska, Piotr Ochodzki, Linda Kærgaard Nielsen, Dorota Walentyn-Góral, Łukasz Stępień

**Affiliations:** 1 Department of Plant Pathology, Plant Breeding and Acclimatization Institute–National Research Institute, Radzików, 05-870 Błonie, Poland; p.ochodzki@ihar.edu.pl (P.O.); d.walentyn-goral@ihar.edu.pl (D.W.-G.); 2 Institute of Plant Genetics, Polish Academy of Sciences, Strzeszyńska 34, 60-479 Poznań, Poland; hwis@igr.poznan.pl (H.W.); lste@igr.poznan.pl (Ł.S.); 3 Sejet Plant Breeding, Nørremarksvej 67, 8700 Horsens, Denmark; lkn@sejet.dk

**Keywords:** *Fusarium* DNA, Fusarium head blight, deoxynivalenol, nivalenol, real time PCR, resistance

## Abstract

Winter wheat lines were evaluated for their reaction to Fusarium head blight (FHB) after inoculation with *Fusarium culmorum* in two field experiments. A mixture of two *F. culmorum* chemotypes was applied (3ADON—deoxynivalenol producing, NIV—nivalenol producing). Different types of resistance were evaluated, including head infection, kernel damage, *Fusarium* biomass content and trichothecenes B (deoxynivalenol (DON), and nivalenol (NIV)) accumulation in grain. The aim of the study was to find relationships between different types of resistance. Head infection (FHB index) and *Fusarium* damaged kernels (FDK) were visually scored. *Fusarium* biomass was analysed using real-time PCR. Trichothecenes B accumulation was analysed using gas chromatography. Wheat lines differ in their reaction to inoculation for all parameters describing FHB resistance. We found a wide variability of FHB indexes, FDK, and *Fusarium* biomass content. Both toxins were present. DON content was about 60% higher than NIV and variability of this proportion between lines was observed. Significant correlation was found between head infection symptoms and FDK. Head infection was correlated with *F. culmorum* biomass and NIV concentration in grain. No correlation was found between the FHB index and DON concentration. Similarly, FDK was not correlated with DON content, but it was with NIV content; however, the coefficients were higher than for the FHB index. *Fusarium* biomass amount was positively correlated with both toxins as well as with the FHB index and FDK. Environmental conditions significantly influenced the DON/NIV ratio in grain. In locations where less *F. culmorum* biomass was detected, the DON amount was higher than NIV, while in locations where more *F. culmorum* biomass was observed, NIV prevailed over DON.

## 1. Introduction

Fusarium head blight (FHB) is a cereal disease caused by fungi of the *Fusarium* genus. These fungi infect cereal heads, causing necrosis of spikelets, kernel infection and damage, and contamination of tissues and grain with *Fusarium* toxins [1]. *Fusarium* species produce many toxins of different chemical groups. As a small grain cereals contaminants, the most important are trichothecenes (primarily deoxynivalenol = DON, nivalenol = NIV, T-2/HT-2 toxins), zearalenone, and moniliformin [2]. Resistance to Fusarium head blight is a composite, quantitative trait. Several types (mechanisms) of resistance were identified [3,4]. They were described as: Type I—resistance to initial infection, type II—to *Fusarium* spread in the spike [5]; type III—to kernel damage, type IV—tolerance against FHB or trichothecene toxins [6]; type V—resistance to accumulation of trichothecene toxins subdivided into: Class 1—by chemical modification [7]; class 2—by hindering of trichothecene synthesis [8].

Resistance levels of type I and type II affect the severity of head infection under field conditions. A high level of type I resistance is relevant in the case of strong pressure of *Fusarium* infection [9]. On the other side, low resistance type II may result in severe head infection despite low infection pressure [10].

It is difficult to estimate resistance type I due to effects of other traits that affect its proper measurement. This trait is the type of flowering [11,12,13]. For wheat and triticale, three types of flowering were described: Open (chazmogamic), when all three anthers escape out of the flower; (cleistogamic), when all the anthers remain inside the flower; and mixed flowering, when one or two anthers remain inside of the flower [14]. As demonstrated in published studies, the least infected were cleistogamic genotypes, whereas the most infected were genotypes of mixed-type flowering [15]. Such observations can be associated with the influence of the presence of anthers containing substances (choline, glycine betaine) stimulating the growth of *Fusarium* [16,17,18,19]. Another factor that may cause difficulties in accurate evaluation is the spread of *Fusarium* to the adjacent spikelets. Based on research available [16,20], about 5–7 days after infection, the pathogen reaches the rachis and grows up or down the head. Consequently, the assessment of the type I resistance requires appropriate methodology. Spraying the heads with spore suspension and observing the number of infected spikelets approximately one week after inoculation is the most commonly used technique [21]. Heads should be inoculated in the same developmental stage (at full anthesis). Occasionally, trichothecene non-producing isolates are also applied, due to their low ability to spread in the head [22].

Precise evaluation of resistance to *Fusarium* spread (type II) is much simpler [23]. Therefore, this type of resistance is most often used in genetic studies on determination of quantitative traits loci (QTL) [24,25,26]. The experimental protocol for the resistance type II is, however, quite labour-intensive because it consists of point inoculation of individual heads [27,28,29]. This is done mainly by injecting the spore suspension to the flower in the middle spikelet of the head. Less commonly, cotton balls soaked in a suspension or fine infested kernels are used (e.g., millet) [25,27,30,31]. The development of the disease is assessed by determining the number of flowers/spikelets with symptoms of necrosis or bleaching. Scoring should be carried out once about 21 days after inoculation, or several times after inoculation in order to accurately determine the rate of the disease progress [10]. It was found that the maximum genetic diversity in terms of resistance occurs about 3 weeks after inoculation with use of DON [25,32]. In susceptible genotypes, the spikelets above the infection point may be dead (wilting), which is a reaction to the phytotoxic effect of DON [33,34].

Assessment of resistance type III determines the proportion of kernels damaged by *Fusarium* in grain sample. This is determined by dividing the sample into factions: Kernels with signs of damage by *Fusarium* (shrivelled, discoloured-white, pink, orange, carmine) and healthy-looking kernels [9]. The percentage of kernels with *Fusarium* damage in the sample is calculated. This type of resistance can also be assessed by analysing the content of ergosterol in the grain. Ergosterol is a component of the cell membranes of fungi [35]. Its contents indicate the amount of mycelium in the grain, which indirectly determines the degree of infection by the *Fusarium* fungi. The amount of mycelium in kernels can also be specified by measuring *Fusarium* DNA content in grain using quantitative PCR (real-time PCR) [36,37,38,39,40]. This method is more accurate because it specifically detects *Fusarium* fungi (or selected *Fusarium* species) in the grain [41,42,43]. Ergosterol specifies the amount of the whole mycobiota, also of fungi outside of *Fusarium* genus [44].

Type IV resistance (tolerance) is determined by measuring the reduction of grain yield caused by Fusarium head blight, followed by a comparison of the degree of kernel damage and concentration of *Fusarium* toxins in the grain [3]. Tolerance to Fusarium head blight (precisely: Tolerance to Fusarium head blight or trichothecenes) occurs when the reduction of yield is low despite a strong infection of heads/kernel damage/accumulation of toxins in the grain. Concerning Fusarium head blight, tolerance is not a favorable trait [4]. High tolerance may result in a high yield of seemingly lightly damaged grain that may be contaminated with *Fusarium* toxins.

The type V resistance is evaluated by quantifying the content of *Fusarium* toxins in the grain [8]. Different methods are applied, such as immunoenzymatic tests or the more precise chromatographic techniques (gas chromatography, liquid chromatography), including the usage of advanced detectors, such as mass spectrometers. In the study of resistance involving chemical modification of the toxins, the amount of ‘masked mycotoxins’ is also determined [45]. These are toxins undergoing glycosylation, which results in the formation of compounds non-toxic to plants, e.g., DON-3-glucoside and zearalenone 14-glucoside. In research on resistance involving blocking the synthesis of toxins, it is important to determine the content of compounds with antioxidant activity, such as phenolic compounds, phenolic acids, carotenoids, or peptides [46].

In this study, we evaluated the reaction of winter wheat breeding lines to Fusarium head blight. Plants were inoculated with *F. culmorum* isolates in field trials. We scored the characters describing different types of resistance, including head infection (types I and II), kernel damage (type III), *Fusarium* biomass concentration in kernels (type III), and trichothecenes group B (DON, DON acetyl derivatives, NIV) accumulation in grain (type V). Results were analysed to find relationships between different characters. As we used mixed inoculation with two chemotypes of *F. culmorum* (3ADON and NIV), we studied the proportions of both toxins in the grain in two experimental environments as well as the relationships between FHB visual assessment parameters and toxin contents.

## 2. Results

Winter wheat lines were inoculated with *F. culmorum* isolates in two field trials. Head infection was scored as the FHB index. After the harvest, the proportion of *Fusarium* damaged kernels (FDK) was assessed. Grain was analysed for the concentration of *F. culmorum* DNA expressing the amount of *F. culmorum* biomass. Finally, the concentration of *Fusarium* toxins belonging to group B of trichothecenes were analysed.

The average FHB index amounted to 11.1% in wheat grown in Cerekwica, ranging from 2.0% to 41.4%. In wheat grown in Radzików, the FHB index was 25.7%, ranging from 9.3% to 48.0%. The overall FHB index for the two locations was 18.4% (Table 1). The average FDK for grain from Cerekwica was 15.2%, ranging from 3.9% to 29.9%. The FDK for grain from Radzików was more than 2-fold higher and amounted to 38.9%, at a range of 10.1–86.5%. The overall FDK proportion for the two locations was 27.0% (Table 1).

*Fusarium culmorum* biomass was detected in all grain samples. The average concentration for Cerekwica was 22.2 pg of *F. culmorum* DNA per one ng of wheat DNA. It ranged from 2.1 to 66.7 pg/ng. The average from Radzików was 2-fold higher and amounted to 45.8 pg/ng with a range from 10.1 to 134.4 pg/ng. It directly corresponds to a much higher FDK in Radzików. Overall, the *Fusarium* biomass concentration for the two locations was 34.0 pg/ng (Table 1).

Presence of DON, 3AcDON (3-acetyl-DON), and NIV was confirmed in all wheat grain samples. Amount of 3AcDON was low with the average at 0.081 mg/kg (0.008–0.297 mg/kg). It was about 2.8% of the average DON amount. DON and 3AcDON amounts correlated highly significantly (*r* = 0.913) so they were further shown as the total amount of DON and 3AcDON. No 15-acetyl-DON was detected.

The average amount of DON (+3AcDON) in grain in Radzików was 1.390 mg/kg and it was 3-fold lower than in Cerekwica (4.418 mg/kg). Similarly, the variation range was wider in Cerekwica (0.100–14.427 mg/kg) than in Radzików (0.282–3.103 mg/kg). Average NIV concentration was 2.578 mg/kg in Radzików, with a range of 0.336–8.820 mg/kg, and 3-fold higher than in Cerekwica (0.982 mg/kg, with a range of 0.044–3.318 mg/kg). Overall, the amount of DON + 3AcDON for the two locations was 2.904 mg/kg (Table 1).

To summarize, concentrations of trichothecenes B (DON + 3AcDON + NIV) in wheat grain in both localities were similar—5.401 mg/kg in Cerekwica and 3.968 mg/kg in Radzików. However, a higher amount of *F. culmorum* biomass found in grain in Radzików led to a higher biomass/trichothecenes ratio in this environment (12.6), compared to Cerekwica (5.6). Regarding the individual toxins, the biomass/DON ratio was low in Cerekwica (8.0) and high in Radzików (34.0). The biomass/NIV ratio was lower in Radzików (22.4) than in Cerekwica (37.1). The overall amount of trichothecenes B for the two locations was 4.684 mg/kg (Table 1).

The wheat lines differed in their reaction to *F. culmorum* inoculation for all parameters describing FHB resistance (Table 1). The most resistant to head infection and kernel damage was line MHR 2. This line also showed the lowest FDK and amount of *F. culmorum* biomass in the grain. Line MHR 2 also displayed high resistance in terms of trichothecenes concentration. However some lines accumulated lower amounts of DON (MHR 5, STH 1, SMH 2) or NIV (MHR 5, STH 8), despite higher *Fusarium* infection. This resulted in a low biomass/trichothecenes ratio (4.7) for MHR 2 compared with other low toxin contaminated lines, e.g., MHR 5 (26.8), STH 1 (13.3), or SMH 2 (19.2).

The highest head infection was seen in line STH 2 (Table 1). This line also had a high FDK proportion. However, some lines of lower infected heads had similar FDK–STH 4 (57.8%) and DANKO 1 (50.1%).

**Figure 1:**
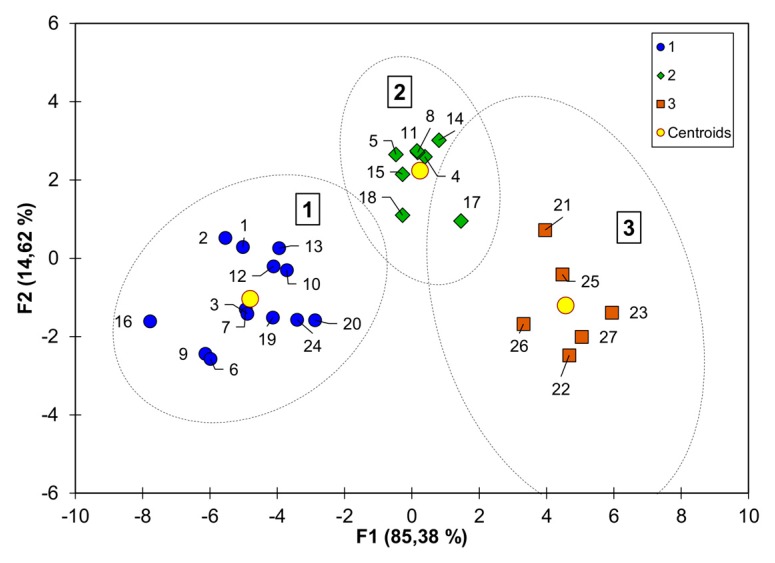
Canonical discriminant analysis of Fusarium head blight index, *Fusarium* damaged kernels, and concentrations of biomass of *Fusarium culmorum* and mycotoxins–DON (+3AcDON) and NIV in grain of 26 winter wheat lines and cultivar ‘Tonacja’. Classes (1, 2, 3) defined by k-means analysis. Line numbers correspond to those in Table 1.

K-means analysis grouped lines based on their FHB resistances of different types (Figure 1, Table 2).

The biomass/trichothecenes ratio was the lowest in the group of susceptible lines (class 3); it was, however, not very different from the ratios for the other groups (Table 2).

**Figure 2:**
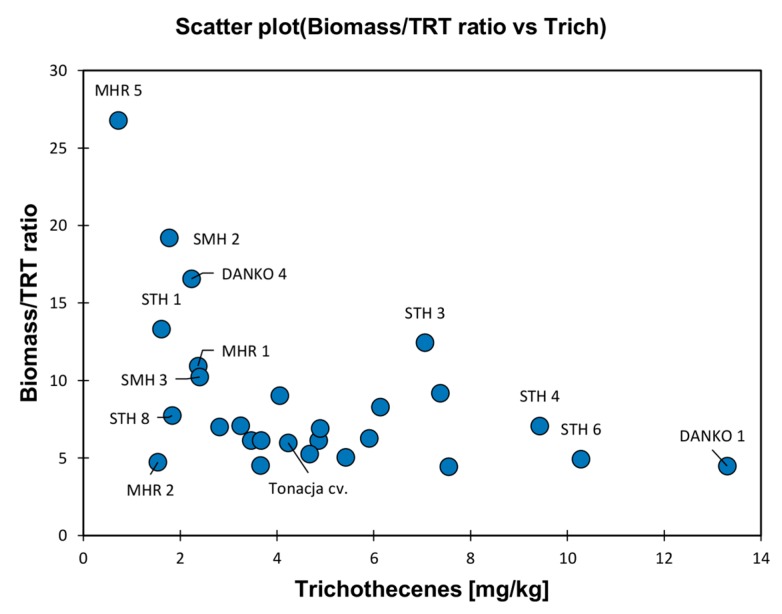
Relationship between concentration of trichothecenes (DON + 3AcDON + NIV) in grain and the *F. culmorum* biomass/trichothecenes (TRT) ratio for 27 winter wheat lines.

The majority of lines showed biomass/trichothecenes ratio values between 5 and 10 (Figure 2). However, five lines with higher values were identified. Four of them accumulated low amounts of trichothecenes B (STH 1, DANKO 4, SMH 2, MHR 5) while one line (STH 3) accumulated more trichothecenes and contained the highest concentration of *F. culmorum* biomass in grain.

A significant linear relationship was found between head infection (FHBi) and amount of *Fusarium* damaged kernels (Table 3). Head infection was also positively correlated with *F. culmorum* biomass and NIV concentration in grain. No significant correlation was found between FHBi and DON concentration. A proportion of *Fusarium* damaged kernels correlated well with *F. culmorum* biomass and NIV concentration. The coefficient for FDK vs. DON was insignificant. The *F. culmorum* biomass amount correlated significantly with the concentration of both mycotoxins; however, the value of the coefficient for DON was much lower than for NIV. The biomass/trichothecenes ratio correlated positively with head infection, kernel damage, and *F. culmorum* biomass. The correlation coefficient ratio vs. DON was significant and negative, whereas there was no correlation with NIV.

As the differences in the relationship of DON and NIV versus FHB resistance traits (FHBi, FDK, *F.c.* biomass) were observed, data from the two trials were analysed separately. A significant correlation was found between the FHBi and DON concentration in Radzików, but not in Cerekwica (Table 4). Coefficients of correlations for FHBi vs. *F. culmorum* biomass and NIV were twice as high in Radzików than in Cerekwica. Regarding the FDK, correlations with DON and NIV in both environments were significant. However, the coefficient of correlation for FDK vs. DON was higher in Cerekwica, while the coefficient of correlation for FDK vs. NIV was higher in Radzików than in Cerekwica. The same was true for the *F. culmorum* biomass.

**Figure 3:**
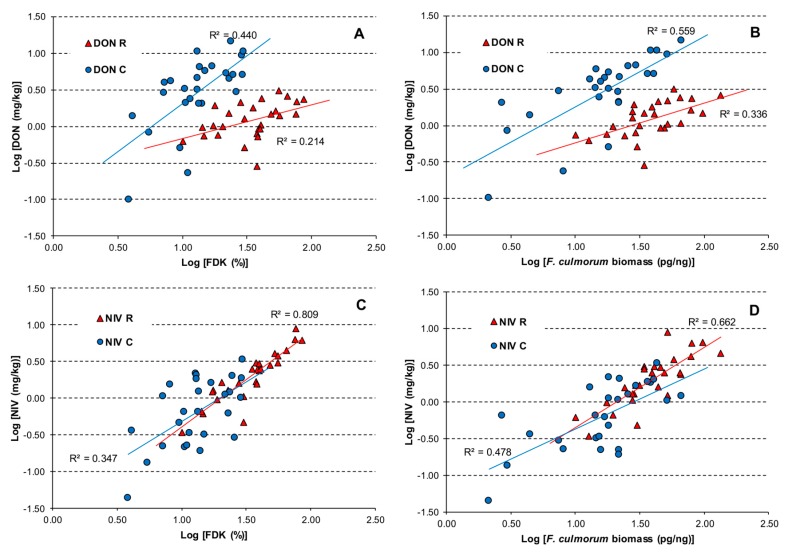
Relationships between FDK and concentration of DON (+3AcDON) (**A**) and NIV (**C**) and the amount of *F. culmorum* biomass and concentration of DON (+3AcDON) (**B**) and NIV (**D**) in grain of wheat lines in Cerekwica (C) and Radzików (R).

To explain the differences between DON and NIV relations with other resistance traits, we analysed the linear regression between them. Figure 3 present the linear regression of FDK and *F. culmorum* biomass versus DON concentration and NIV concentration in two experimental environments. The DON concentration in grain in Cerekwica was higher than in Radzików for the majority of the samples despite the FDK or amount of *F. culmorum* biomass (Figure 3A,B). The NIV concentration increased with the increase of FDK or *F. culmorum* biomass in grain (Figure 3C,D). However, it was noticeable that in Cerekwica, some lines with similar FDK values accumulated different amounts of NIV. It was less expressed for *F. culmorum* biomass.

Hence, the low correlation of the DON amount with phenotypic characters (FHBi, FDK) and *F. culmorum* biomass and high values of coefficients for NIV (Table 3).

## 3. Discussion

In both locations, inoculations were effective even though weather conditions during flowering were unfavourable for *Fusarium* infection. In Cerekwica, the influence of drought conditions was compensated by application of mist irrigation post inoculation. In Radzików, evening inoculations were sufficient for infection. However, weather conditions in the following weeks were unfavourable for the appearance of FHB symptoms in Cerekwica, which resulted in differences in FHB indexes, FDK values, and DON content. Higher FDK values in Radzików can be explained by the incidence of low and frequent rainfalls in Radzików in July. In comparison, Cerekwica was affected by drought in July, slowing down the development of FHB. The concentration of DON in grain was relatively low, even though the proportion of *Fusarium* damaged kernels was high. On the contrary, NIV concentration was high, mostly in Radzików. The amount of NIV in wheat grain samples in Radzików was 2-fold higher than the DON amount.

The results show that the environmental conditions significantly influenced the ratio of the 3ADON/NIV chemotype and affected the shift between DON/NIV production. It appears that in Radzików, conditions were more favourable for NIV chemotype development. The *F. culmorum* NIV chemotype outcompeted the DON chemotype in Radzików, and in Cerekwica, the situation was the opposite. We can presume that we are dealing with competition between two chemotypes of the same species competition between the two chemotypes of the same species. Van der Ohe and Miedaner [47] described the competition between *F. culmorum* and *F. graminearum* species. They also included one isolate of *F. graminearum* NIV chemotype. This isolate showed similar pathogenicity to other DON chemotype isolates. Mixture of DON+NIV chemotypes had stable pathogenicity (FHB rating), but differed in toxin production across environments. The concentration of NIV after mixture inoculations was not determined, hence the concentration in the first experimental year is unknown. The results showed that the NIV chemotype isolate dominated this year.

Only the total *Fusarium* biomass was analysed, so the ratio of 3ADON/NIV chemotypes biomass in single grain samples is unknown. This issue could be resolved by quantitative analysis of 3ADON and NIV chemotypes biomass of *F. culmorum* [48]. The NIV chemotype is generally considered less aggressive than DON (3ADON, 15ADON) chemotypes [49,50]. However, our results showed that it could produce considerable amounts of NIV even in mixture with a more aggressive isolate of the 3ADON chemotype. This is evident in lines, PHR 1, DANKO 3, and STH 3, in which amounts of DON and NIV were similar, and line STH 2, which accumulated more NIV than DON in grain (Table 1).

Linear relationships of *F. culmorum* biomass vs. NIV were significant in both locations. This was probably, as mentioned above, the result of different mycotoxin (DON vs. NIV) profiles in the two locations. These differences are shown in the figures. Figure 3D shows a clear effect of the increasing amount of *F. culmorum* biomass on NIV content. Figure 3B shows a weak linear effect of the increasing amount of *F. culmorum* biomass on DON content for all samples. However, linear relationships were highly significant in the two locations separately. NIV content in grain was low in Cerekwica, which was in accordance with the lower amount of *F. culmorum* biomass than that observed in Radzików. It seems that the DON/NIV ratio depended strongly on environmental conditions. As in Cerekwica, conditions after inoculations were drier than in Radzików; we can presume that they were favourable for a more aggressive 3ADON chemotype. Whether or not it was caused by competition of two *F. culmorum* isolates of different chemotypes is unknown and the issue needs further research. The DON vs. NIV relationship was significant, but weak for data from both locations differing in environmental conditions. However, it was highly significant to separate data from locations. DON and NIV are both FHB aggressiveness factors and cause similar symptoms on wheat heads [51]. Despite this fact, differences in the detoxification mechanism of both toxins were found. Lemmens et al. [40] suggested that different genes in the *Fhb1* gene cluster may be involved in resistance to these toxins. NIV is less toxic to wheat plants than DON and NIV isolates appear to be less aggressive [52,53]. On the contrary, NIV is more toxic to humans and animals [54]. Amounts of NIV detected in agricultural products are lower than amounts of DON, but both mycotoxins can co-occur and pose a threat to consumers when their total amount is above maximum limits [55].

Comparing the correlation coefficients between different parameters of FHB assessment (FHBi, FDK, *F. culmorum* biomass) and toxin content, we find that biomass is the best predictor of the amount of trichothecene toxins. The FHB index correlated with DON and NIV in Radzików, but not with DON in the different environment in Cerekwica and only weakly with NIV. It resulted in a lack of correlation with DON and the sum of trichothecenes. Higher and significant coefficients were observed for FDK in separate samples from locations; however, for aggregated samples, the coefficient for DON was insignificant. Mesterhazy [3] showed such variability of relationships in multi-year experiments.

Brunner et al. [56] studied relationships between visual scoring, infection determined with real-time PCR, and toxin (DON + D3G) content in grain for wheat inoculated with *F. graminearum* and *F. culmorum*. They found that evaluation of FHB resistance based on DNA quantification is more reliable than on visual scoring, especially for lines that are more resistant. They observed low compliance of visual scoring with toxins and DNA for *F. graminearum*, but high for *F. culmorum*. We observed the same for results from Radzików, where FHB severity and toxin content was high, but not for Cerekwica, where FHB infection was much lower. Horevaj et al. [57] obtained similar results for *F. graminearum*. Visual assessment of FHB severity showed lower correlations with DON content than FDK and *F. graminearum* biomass. They tested FHB resistant lines and susceptible check and observed very low FHB severity for resistant lines and high for check. Whereas, for FDK and biomass, the resistant were evenly distributed and some lines showed values close to the susceptible check.

A stronger relationship between *Fusarium* biomass and DON content than between the FHB index, FDK, or other visual scoring parameters has been confirmed by other authors [37,58,59,60]. Rossi et al. [60] studied FHB resistance parameters in bread wheat and durum wheat inoculated with *F. culmorum* and *F. graminearum*. They found stable relationships between *Fusarium* biomass and DON content for different species of wheat and *Fusarium* in different environments.

The above results confirm that selection for FHB resistance based only on visual scoring does not necessarily ensure low levels of trichothecenes in grain of selected lines. It is less problematic in wheat, where in most cases, the severity of symptoms of head infection significantly correlates with toxin content (in experimental conditions) [61,62]. However, in triticale, a weak relationship between head infection and toxin content in grain is frequently observed, and in many cases, between the severity of kernel damage and toxins [63,64,65,66,67].

## 4. Conclusions

Environmental conditions significantly influenced the DON/NIV ratio in grain when wheat heads were inoculated with a mixture of two chemotypes of *F. culmorum*-3ADON and NIV. In the location where lower *F. culmorum* biomass was detected, the amount of DON was higher than NIV. While in the location where *F. culmorum* biomass concentration was higher, NIV prevailed over DON.

The relationship between *Fusarium* biomass and trichothecenes B content in grain was stronger than between visually scored head infection (FHB index) and the proportion of damaged kernels (FDK). We found differences in the relationship between *Fusarium* biomass vs. DON or NIV. For NIV, increasing the amount of *Fusarium* biomass in grain from both locations resulted in an increase in the amount of NIV. Whereas for DON, the same amounts of *Fusarium* biomass in two locations resulted in different amounts of DON.

## 5. Materials and Methods

### 5.1. Fusarium culmorum Isolates

Inoculum for field experiments was produced from three *Fusarium culmorum* isolates. Two of them (KF846, ZFR112) were 3ADON chemotypes and one (KF350) was the NIV-chemotype. These produced DON and 3AcDON-KF846, ZFR112 as well as NIV-KF350 [68]. Isolates of 3ADON chemotype were isolated originally from wheat heads collected in Radzików, Poland [68,69]. The isolate, KF350 (NIV chemotype), was isolated from wheat heads collected in the Netherlands [70].

Species identity of the strains was confirmed using species-specific molecular markers [71,72]. Moreover, a portion of fungal *TEF*-1α gene was amplified, sequenced, and analysed, as described previously [73]. Gene-specific markers were used to identify *TRI5, TRI13*, and *PKS13* genes, characteristic for trichothecene and zearalenone chemotypes of *Fusarium* spp. Sequencing of the amplified DNA fragments was conducted to confirm the chemotypes of the strains. All primer sequences, PCR amplification, and sequencing protocols were described in a previous work [73].

*Fusarium culmorum* isolates were incubated on autoclaved wheat grain in 300 mL Erlenmeyer glass flasks for 7 days in darkness at 18 °C, and next exposed to near ultraviolet light (350 nm) with a 16-h photoperiod at 15 °C for about 21 days. Flasks were shaken thoroughly every day to prevent sticking of the grain overgrown with the mycelium. Grain with visible sporulation of *F. culmorum* on the kernel surface was dried and stored in at 4 °C until use. Prior to the inoculation, grain with *F. culmorum* mycelium and conidia were suspended in distilled water for 1 h and filtered through two layers of cheesecloth to obtain a conidial suspension without mycelium. Concentrations of the suspensions from all the isolates were adjusted to 5 × 10^5^ spores/mL with a hemocytometer. Equal volumes of conidial suspensions of three isolates were mixed.

### 5.2. Field Experiments

Resistance of 26 breeding lines and cultivar ‘Tonacja’ of winter wheat was evaluated. ‘Tonacja’ is one of the most frequently grown wheat cultivars in Poland and is medium-resistant to FHB. Wheat lines were sown in two field trials located in Radzików near Warsaw (GPS coordinates 52.212612, 20.633111) and in Cerekwica near Poznań (GPS coordinates 52.522579, 16.688624) in 2008. Field trials were arranged in a randomized complete block design. Wheat was sown at 1 m^2^ plots in four replicates. Three replicates were inoculated; the fourth replicate was left uninoculated and served as a check of natural infection. Fungicides were not applied to the experimental plots.

Wheat heads were inoculated at the anthesis stage with conidial suspension of *F. culmorum.* The inoculation rate was about 100 mL of suspension per plot (1 m^2^). Plots were inoculated separately at the beginning of anthesis (61 BBCH), and inoculation was repeated about three days later at full anthesis (65 BBCH). Flowering time in Cerekwica, located in western Poland, was about one week earlier than in Radzików. Full anthesis stage was on average in Cerekwica, on June 1, and in Radzików, on June 8. In Radzików, plots were inoculated in the evening, when the relative air humidity increased. In Cerekwica, plots were mist-irrigated for 3 days after inoculation.

The mean percentage of blighted spikelets per infected head (FHB severity) and the percentage of infected heads per plot (FHB incidence) were scored. Disease severity was scored only on heads on a plot showing FHB symptoms. Fusarium head blight index (FHBi) was calculated from the FHB severity and FHB incidence using the following formula:

(1) $${\mathrm{FHB}}_{i}=\left({\mathrm{FHB}}_{\mathrm{incidence}}\times {\mathrm{FHB}}_{\mathrm{severity}}\right)/100$$

Fusarium head blight was assessed about 21 days after the last inoculation. At harvest, 30 heads were collected manually from each plot and threshed with a laboratory thresher at low wind speed to prevent loss of low-weight infected kernels. The number of *Fusarium* damaged kernels (FDK) was visually evaluated by dividing the grain sample into two categories: Healthy kernels and infected kernels showing different levels of damage [29]. Next, whole grain samples were roughly milled with a laboratory grinder and stored at −20 °C until further analyses of DNA and mycotoxins.

### 5.3. DNA Extraction

Five grams of milled grain was ground powdered in liquid N_2_ with eight steel balls using a homogenizer Geno/Grinder 2000 (OPS Diagnostics, Bridgewater, NJ, USA). DNA was extracted from 100 mg of grain powder using a slightly modified CTAB (cetyl trimethylammonium bromide) method (http://gmo-crl.jrc.it/summaries/NK603-WEB-Protocol%20Validation.pdf) [42]. The DNA samples were finally purified with a DNeasy Plant Kit (QIAGEN, Stockach, Germany). The manufacturer’s procedure was applied.

*Fusarium culmorum* isolate 9560 was used for standard curves [74]. The isolate was grown on Potato Dextrose Agar (PDA) medium covered with sterile polyethylene circles. PDA plates were incubated at 22 °C with a 12 h photoperiod for one week. Next, mycelium was scraped from the polyethylene surface with a spatula and ground. The same method as for grain was used for mycelium grinding and *F. culmorum* DNA extraction.

### 5.4. Fusarium culmorum DNA Analysis

The concentration of *F. culmorum* DNA in grain of wheat lines was analysed using quantitative real-time PCR according to the methodology developed by Nicolaisen et al. [42]. Two pairs of primers based on TEF-1α genes were applied-primers specific for *F. culmorum* and for plant DNA (wheat). (Table 5).

Quantitative real-time PCR was carried out using SYBR Green method described in detail by Nielsen et al. [48,74]. PCR was performed on a 7900HT Sequence Detection System (Applied Biosystems, Foster City, CA, USA).

Standard curves for the *F. culmorum* species and wheat were made of a fivefold dilution series using fungal DNA from pure cultures (described above) as well as wheat DNA. The amount of *F. culmorum* DNA was calculated from the cycle threshold (*Ct*) values using the standard curve.

As SYBR Green (DNA-binding fluorescent dye) binds to all double-stranded DNA and the results for each sample were evaluated by examining the dissociation curve and Ct value. The plant assay was used to calculate a relative measurement for *Fusarium* biomass in each sample, which was expressed as picograms of fungal DNA per micrograms of plant DNA according to Nicolaisen et al. [42] and Nielsen et al. [74].

### 5.5. Analysis of Mycotoxins

The analysis of mycotoxins was based on extraction with aqueous acetonitrile, purification with MultiSep^®^ 227 Trich+ columns (Romer Labs Inc., Union, MO, USA), and GC-ECD analysis method, according to Weingaertner et al. [75] with modifications.

Mycotoxins were extracted from 5 g of ground grains using 25 mL of an aqueous solution of acetonitrile (acetonitrile:water, 84:16, *v*/*v*). Samples were shaken on the laboratory shaker overnight, centrifuged (3000 rpm/min, 5 min), and 6 mL of the extract was purified with MycoSep^®^ 227 Trich+ columns (Romer Labs Inc., Union, MO, USA). Internal standard solution (α-chloralose, 1 mL, 1 µg/mL in acetonitrile) was added to 4 ml of purified extract and the solution was evaporated to dryness in the stream of air. Mycotoxins were derivatized to the trimethylsilyl derivatives with 75 µL of derivatizing agent, Sylon BTZ (BSA + TMCS + TMSI, 3:2:3, Supelco), and heated 30 min in 60 °C. After dissolution of sample in 1 mL of isooctane, the excess of derivatizing agent was decomposed and removed with water. The organic layer was transferred to an autosampler vial and 1 microliter of solution was injected on GC.

Content of the trichothecenes of group B in the grain (DON, 3AcDON, 15AcDON, NIV) was analysed using the gas chromatography technique. Gas chromatograph SRI 8610C (SRI Instruments, Earl St. Torrance, CA, USA), equipped with a splitless injector, ^63^Ni electron capture detector (ECD) (VICI Valco Instruments, Schenkon, Switzerland), HT300A autosampler (HTA S.R.L., Brescia, Italy), HG 2200 (CLAIND srl, Tremezzo, Italy) hydrogen generator, BGB-5MS 30 m × 0.25 mm × 0.25 µm column (BGB Analytik Vertrieb GmbH, Rheinfelden, Germany) and PeakSimple data processing program were used.

The carrier gas was hydrogen, adjusted to pressure 12 psi, with nitrogen as a make-up gas at 60 mL/min. Elution was carried out in the temperature gradient: Initial temperature was 170 °C, increased to 250 °C at 5 °C/min., and increased from 250 °C to 300 °C at 10 °C/min., followed by a holding time of 5 min., and decreased to 170 °C. Individual compounds were identified by comparing the retention time of these with the retention times of the pure standards of mycotoxins (Biopure). The injection port and detector operated at 250 °C and 300 °C, respectively. The concentration of mycotoxins was established based on the calibration curve, using α-chloralose (Sigma-Aldrich sp. z o.o., Poznań, Poland) as the internal standard.

Preliminary studies on the analytical method (data not shown) revealed the repeatability (RSD) of the method, 7.2% for DON and 10.6% for NIV. Experiments on wheat meal spiked with pure mycotoxins (1000 µg/kg of each) showed recovery of DON—93% and NIV—72%. All results were corrected for recovery.

### 5.6. Statistical Analysis

Analysis of variance of the FHB index and FDK data (including Tukey’s pairwise comparison at the level of significance *p* = 0.05) was accomplished using the GLM (general linear model) procedure of SAS 9.2 package (SAS Institute Polska, Warszawa, Poland, 2008). Data were analysed separately for two locations and combined with location as the independent effect.

The relationships between the results for the FHB index, FDK, and *Fusarium* biomass and toxins were explored by Pearson correlation tests. Prior to analysis, all data were log transformed to stabilize variances. Wheat lines were grouped according to their resistance to FHB using the k-means clustering method. Groups of lines were created based on their resistance characterized by the FHB index, FDK, amounts of *F. culmorum* DNA, DON, and NIV. Trace (W) classification criterion was applied. This criterion is sensitive to effects of scale; thus, all variables were standardized prior to the analysis.

The correlation and k-means analysis were performed using Microsoft^®^ Excel 2010/XLSTAT©-Pro (Version 2015.2.02.18135, Addinsoft, Inc., Brooklyn, NY, USA).

## Figures and Tables

**Table 1:** Fusarium head blight resistance of 27 winter wheat lines inoculated with *F. culmorum* isolates in two locations—Radzików and Cerekwica.

No.	Line ^1^	Fusarium Head Blight Index (%)	*Fusarium* Damaged Kernels (%)	*F.c.* Biomass (pg/ng)	DON ^2^ (mg/kg)	NIV (mg/kg)	TRT ^3^ (mg/kg)	Biomass/TRT Ratio
1	MHR 5	15.3	20.7	19.3	0.371	0.348	0.719	26.8
2	MHR 2	6.9	9.4	7.3	1.057	0.482	1.539	4.7
3	STH 1	24.1	24.0	21.4	0.608	1.001	1.609	13.3
4	SMH 2	12.9	22.0	34.1	0.576	1.198	1.774	19.2
5	STH 8	13.2	10.8	14.2	1.498	0.335	1.833	5.3
6	DANKO 4	18.1	15.8	37.0	1.532	0.702	2.234	16.6
7	MHR 1	11.1	24.1	25.9	1.428	0.939	2.367	10.9
8	SMH 3	15.4	22.6	24.6	0.870	1.533	2.403	10.2
9	STH 5	17.5	13.0	19.7	1.805	1.007	2.812	7.0
10	PHR 2	17.8	15.4	23.0	2.100	1.148	3.248	7.1
11	SMH 1	17.2	24.4	21.3	1.911	1.557	3.468	6.1
12	STH 7	10.8	11.2	16.5	2.566	1.094	3.660	4.5
13	DANKO 5	19.1	26.7	22.5	2.840	0.832	3.672	6.1
14	SMH 4	22.4	33.6	36.6	2.673	1.383	4.056	9.0
15	Tonacja cv.	12.3	27.8	25.3	2.717	1.515	4.232	6.0
16	PHR 3	13.2	26.3	24.5	3.005	1.667	4.672	7.7
17	PHR 1	12.5	34.6	29.9	2.286	2.573	4.859	6.1
18	MHR 3	23.5	30.3	33.8	3.096	1.795	4.891	6.9
19	MHR 4	17.6	15.6	27.3	4.173	1.249	5.422	5.0
20	DANKO 2	9.5	22.5	37.1	4.304	1.602	5.906	6.3
21	DANKO 3	19.0	32.9	50.8	3.068	3.064	6.132	8.3
22	STH 3	28.8	45.1	87.8	3.804	3.253	7.057	12.4
23	STH 2	44.7	52.8	67.6	3.262	4.110	7.372	9.2
24	PHR 4	16.0	18.0	33.5	6.005	1.540	7.545	4.4
25	STH 4	24.6	57.8	66.6	5.811	3.612	9.423	7.1
26	STH 6	30.6	42.8	50.7	6.742	3.534	10.276	4.9
27	DANKO 1	22.0	50.1	59.6	8.303	4.998	13.301	4.5
	Mean	18.4	27.0	34.0	2.904	1.780	4.684	8.7

^1^ Lines sorted according to total concentration of trichothecenes B; ^2^ total amount of DON and 3AcDON; ^3^ TRT = trichothecenes, sum of DON, 3AcDON, and NIV.

**Table 2:** Average values and ranges for classes created by k-means cluster analysis of FHB index, FDK, *F. culmorum* biomass, and DON and NIV concentrations in grain of 27 wheat lines.

Class	No. of Lines	FHBi (%)	FDK (%)	*F.c.* Biomass (pg/ng)	DON ^1^ (mg/kg)	NIV (mg/kg)	Biomass/TRT ^2^ Ratio
1	13	15.2 (6.9–24.1)	17.5 (9.4–26.7)	23.4 (7.3–37.1)	2.330 (0.371–6.005)	0.944 (0.335–1.602)	9.3 (4.4–26.8)
2	8	16.2 (12.3–23.5)	27.7 (22.0–34.6)	28.8 (21.3–36.6)	2.142 (0.576–3.096)	1.653 (1.198–2.573)	8.6 (5.3–19.2)
3	6	28.3 (19.0–44.7)	46.9 (32.9–57.8)	66.5 (50.7–87.8)	5.165 (3.068–8.303)	3.762 (3.064–4.998)	7.7 (4.5–12.4)
Total/mean	27	18.4	27.0	34.0	2.904	1.780	8.7

^1^ Total amount of DON and 3AcDON; ^2^ TRT = trichothecenes, sum of DON, 3AcDON, and NIV

**Table 3:** Correlations between FHB index (FHBi), FDK, *F. culmorum* biomass (*F.c.* biomass), concentration of DON, NIV, and sum of trichothecenes in grain (TRT), and the ratio of fungal biomass per amount of DON + NIV produced (Biomass/TRT) for data for 27 winter wheat lines from two locations (54 samples). Variables log transformed.

Variables (*n* = 54)	FHBi (%)	FDK (%)	*F.c.* Biomass (pg/ng)	DON ^1^ (mg/kg)	NIV (mg/kg)	TRT (mg/kg)
FDK [%]	**0.780**					
*F.c.* biomass [pg/ng)	**0.648**	**0.792**				
DON [mg/kg)	−0.173	0.102	**0.319**			
NIV [mg/kg)	**0.672**	**0.802**	**0.801**	**0.291**		
TRT [mg/kg)	0.191	**0.509**	**0.649**	**0.858**	**0.704**	
Biomass/TRT ratio	**0.545**	**0.339**	**0.421**	**−0.641**	0.117	**−0.418**

^1^ Total amount of DON and 3AcDON; values displayed in bold are significant at the 0.05 significance level.

**Table 4:** Correlations between FHB index (FHBi), FDK, *F. culmorum* biomass (*F.c.* biomass), and concentration of DON and NIV in grain of 27 winter wheat lines in Cerekwica (C) and Radzików (R). Variables log transformed.

Variables	FHBi C	FHBi R	FDK C	FDK R	*F.c.* Biomass C	*F.c.* Biomass R	DON ^1^ C	DON ^1^ R	NIV C
FHBi R	**0.464**								
FDK C	**0.578**	**0.610**							
FDK R	0.146	**0.670**	**0.491**						
*F.c.* biomass C	**0.381**	**0.388**	**0.672**	0.285					
*F.c.* biomass R	0.259	**0.744**	**0.559**	**0.796**	0.254				
DON ^1^ C	0.212	0.243	**0.663**	0.185	**0.748**	0.174			
DON ^1^ R	0.102	**0.514**	**0.523**	**0.462**	0.374	**0.580**	**0.482**		
NIV C	**0.428**	0.268	**0.589**	0.208	**0.692**	0.194	**0.750**	**0.509**	
NIV R	0.125	**0.711**	**0.484**	**0.899**	**0.328**	**0.813**	**0.383**	**0.540**	0.280

^1^ Total amount of DON and 3AcDON; values displayed in bold are significant at the 0.05 significance level.

**Table 5:** Sequences and names of *F. culmorum* and plant EF1α gene specific primers [31].

Target	Primer Name	Sequence (5′–3′)
*F. culmorum*	FculC561 fwd FculC614 rev	CACCGTCATTGGTATGTTGTCACT CGGGAGCGTCTGATAGTCG
Plant TEF-1α	Hor1f Hor2r	TCTCTGGGTTTGAGGGTGAC GGCCCTTGTACCAGTCAAGGT
